# Ovine progressive pneumonia provirus levels are unaffected by the prion *171R *allele in an Idaho sheep flock

**DOI:** 10.1186/1297-9686-41-17

**Published:** 2009-01-22

**Authors:** Robert D Harrington, Lynn M Herrmann-Hoesing, Stephen N White, Katherine I O'Rourke, Donald P Knowles

**Affiliations:** 1 Animal Disease Research Unit, Agricultural Research Service, US Department of Agriculture, Pullman, WA 99164-6630, USA; 2 Department of Veterinary Microbiology and Pathology, Washington State University, Pullman, WA, 99164-7040, USA; 3 Department of Comparative Medicine, University of Washington, Seattle, WA 98195-7190, USA; 4 Center for Integrated Biotechnology, Washington State University, Pullman, WA 99164, USA

## Abstract

Selective breeding of sheep for arginine (*R*) at prion gene (*PRNP*) codon 171 confers resistance to classical scrapie. However, other effects of *171R *selection are uncertain. Ovine progressive pneumonia/Maedi-Visna virus (OPPV) may infect up to 66% of a flock thus any affect of *171R *selection on OPPV susceptibility or disease progression could have major impact on the sheep industry. Hypotheses that the *PRNP 171R *allele is 1) associated with the presence of OPPV provirus and 2) associated with higher provirus levels were tested in an Idaho ewe flock. OPPV provirus was found in 226 of 358 ewes by quantitative PCR. The frequency of ewes with detectable provirus did not differ significantly among the *171QQ*, *171QR*, and *171RR *genotypes (p > 0.05). Also, OPPV provirus levels in infected ewes were not significantly different among codon 171 genotypes (p > 0.05). These results show that, in the flock examined, the presence of OPPV provirus and provirus levels are not related to the *PRNP 171R *allele. Therefore, a genetic approach to scrapie control is not expected to increase or decrease the number of OPPV infected sheep or the progression of disease. This study provides further support to the adoption of *PRNP 171R *selection as a scrapie control measure.

## Introduction

Scrapie is the prototypical prion disease and one of several described in animals and humans. Accumulation of disease associated prion protein (PrP^Sc^), an abnormally folded form of normal host prion protein (PrP^C^), is central to disease and expression of the host prion gene (*PRNP*) is necessary in pathogenesis [1]. *PRNP *open reading frame (ORF) variants associate with disease incubation time [2] and relative disease susceptibility in sheep [3-7], goats [8-10], elk [11-13], deer [12,14] and humans [15-18].

Polymorphisms in sheep at *PRNP *codons 136 (Alanine/Valine), 154 (Arginine/Histidine), and 171 (Glutamine/Arginine) are involved in scrapie susceptibility (for review see [19]). Codon 171 is an important element of susceptibility in the United States (US) sheep population [6,7]. Sheep homozygous for glutamine at codon 171 (*171QQ*) are highly susceptible to Scrapie, whereas sheep heterozygous (*171QR*) or homozygous (*171RR*) for arginine are highly resistant to classical strains of US Scrapie.

The *PRNP 171Q *allele predominates in US sheep whereas the *171R *allele and *171RR *genotype are less common (the latter two occur at a frequency of about 37% and 16%, respectively [20]). Selective breeding for the *171R *minor allele to produce animals with the *171QR *or *171RR *genotypes is sometimes used as a Scrapie control measure, however the functional consequences of *171R *selection on other traits is uncertain. Genetic selection may have unexpected positive or negative effects as individual genes may have multiple biological roles (pleiotropy) or may be linked to other genes that impact overall biological functions. Uncertainty regarding *PRNP *selection effects (beyond Scrapie resistance) has led to investigation of multiple ovine traits related to reproduction, milk, meat, fiber and genetic diversity. However, *PRNP *selection effects on disease susceptibility (besides Scrapie) has only been studied for *Salmonella *resistance [21].

Ovine progressive pneumonia/Maedi-Visna virus (OPPV) is a monocyte/macrophage tropic lentivirus (a subclass of retrovirus) endemic in many US sheep flocks and causes pneumonia, mastitis, arthritis and encephalitis. One in five sheep are infected based on detection of anti-OPPV serum antibodies and seroprevalence can be as high as 66% in open rangeland environments [22,23]. As many as 76% of OPPV seropositive sheep may develop OPPV related diseases [24]. OPPV quantitative PCR (qPCR) is an alternative method to detect lentivirus and provides both diagnostic and prognostic information [25-27]. The qPCR assay measures the presence and amount of virus that has been reverse-transcribed and integrated into the host genome (provirus). The technique is a useful indicator of disease progression in the study of OPPV because OPPV provirus levels correlate with the severity of pulmonary lesions [28,29].

Scrapie is diagnosed in about one of every 500 culled sheep [20] thus OPPV has much greater prevalence. Uncertainty regarding whether *PRNP *selection would effect OPPV provirus levels can create producer reluctance to the implementation of *171R *selection when OPPV is a more severe flock-health problem. A prion-retrovirus pathogenic relationship of undetermined mechanisms has been observed between PrP^Sc ^and Murine Leukemia Virus (MuLV) [30], PrP^Sc ^and Caprine Arthritis Encephalitis Virus (CAEV) [J Stanton, personal communication], PrP^Sc ^and mastitis presumptively caused by OPPV [31], and influence of PrP^c ^expression on HIV infection [32]. In this study, the following two hypotheses were tested in an Idaho ewe flock: 1) the *PRNP *codon *171R *allele is associated with the presence of OPPV provirus and 2) the *PRNP 171R *allele is associated with higher OPPV provirus levels. This study will help guide producer decisions and it provides information for future prion-retrovirus co-infection studies and advances knowledge of whether *PRNP *selection affects other infectious diseases.

## Methods

### Animals

Three hundred fifty eight ewes were sampled from a flock in southeastern Idaho in which OPPV is endemic and there are no reported cases of scrapie. Animals were cared for under guidelines of the United States Sheep Experimental Station Institutional Care and Use Committee. Breeding was performed without prior selection of prion genotype. The sample set was composed of 117 Columbia, 116 Polypay, and 125 Rambouillet sheep. Ages were three, four, five and six years with 39, 30, 31, and 17 Columbia; 27, 31, 33, and 25 Polypay; and 32, 32, 36, and 25 Rambouillet, respectively.

### Nucleic acid extraction

Peripheral blood leukocytes (PBL) were isolated from whole blood as described [23]. Genomic DNA was extracted from PBL using a commercial kit (Gentra, Minneapolis, Minnesota).

### *PRNP *Genotyping

DNA amplification and sequencing of the ovine *PRNP *ORF was performed similarly to previous experiments using forward primer 5'-GGCATTTGATGCTGACACC-3' and reverse primer 5'-TACAGGGCTGCAGGTAGAC-3' [33]. Reverse primer 5'-GGTGGTGACTGTGTGTTGCTGA-3' was used for standard dideoxynucleotide sequencing. All sequencing was performed at the Laboratory for Biotechnology and Bioanalysis (Washington State University, Pullman, WA). *PRNP *genotypes were analyzed using commercial software (Vector NTI, Invitrogen; Carlsbad, CA or Lasergene Seqman Pro v7.1, DNAstar, Inc, Madison, WI) and codon variants reported by single letter code (*e.g*. glutamine *Q*, arginine *R*, valine *V*, histidine, *H*, leucine *L*, phenylalanine *F*).

### OPPV quantitative PCR

PPV provirus level was determined using a previously described quantitative real-time PCR (qPCR) assay [23]. The OPPV qPCR used primers TMENVCONf 5'-TCA TAG TGC TTG CTATCA TGG CTA-3' and TMENVCONr 5'-CCG TCC TTG TGT AGG ATT GCT-3' (Invitrogen Corporation, Carlsbad, CA) and a Taqman 5'-5'-hexachlorofluorescein-AGC AAC ACC GAG ACC AGC TCC TGC-3' Black Hole Quencher-1 probe (Integrated DNA Technologies, Coralville, IA) targeting the highly conserved transmembrane region within the envelope gene of the North American OPPV strains [34].

### Statistical analyses

Two types of genotypic comparison were made using provirus data and *PRNP *genotype, with a minimum *PRNP *allele frequency of 10% required for analysis. Association between *PRNP *genotype and presence or absence of OPPV provirus was tested using logistic regression models from the logistic procedure of SAS v9.1 (SAS Institute, Cary, NC). Association between *PRNP *genotype and the level of logarithm (base 10)-transformed provirus in OPPV positive animals was tested using the glm procedure in SAS v9.1. In each case the association model included breed as a categorical predictor, age as a linear covariate, the interaction between breed and age, and the *PRNP *genotype of interest. Adjusted odds ratios and 95% confidence interval were calculated for the pair-wise comparison of the frequency of OPPV positive ewes in each *PRNP *genotype. Adjusted mean log-transformed provirus levels were reported with 95% confidence intervals. Stepdown Bonferroni p-value correction [35] was applied separately to each set of analyses.

## Results

### Distribution of *PRNP *genotypes

**Figure 1:**
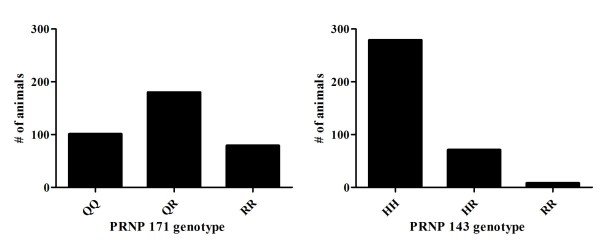
**Number of sheep distributed among *PRNP *genotypes. Left **= codon 171, **Right **= codon 143, **y-axis **= number of animals.

The *PRNP *genotypes were determined as the first step in testing association with the presence of OPPV provirus and OPPV provirus levels. *PRNP *ORF coding variants were identified at codons 101(*Q/R*), 136(*A/V*), 141(*L/F*), 143 (*H/R*), 154 (*R/H*), and 171 (*Q/R*) (Table 1). Of the 358 sheep sampled, 100 (28%) were *171QQ*, 179 (50%) were *171QR *and 79 (22%) were *171RR*, providing a representation of all three genotypes (Fig. 1, left). Examination of the *171R *allele relative to the overall *PRNP *ORF showed that in all animals with the *171RR *genotype there were no other *PRNP *codon variants present. Codon changes at other positions only occurred in animals that had at least one wild type *171Q *allele. Of the 358 sheep, 279 (78%) were *143HH*, 71 (20%) were *143HR *and 8 (2%) were *143RR *(Fig. 1, right). Since codons 143 and 171 had amino acid substitutions with a minor allele frequency of at least 10% they were further analyzed, except for the rare *143RR *genotype. Codons 101, 136, 141, and 154 had a minor allele frequency of less than 10% and therefore these four codons were excluded from further association analysis.

**Table 1:** Distribution of PRNP ORF codon variants among individual breeds and in cumulative sample set

PRNP genotype	Columbia	Polypay	Rambouillet	Total
101QQ	96	115	112	323
101QR	21	1	12	34
101RR	0	0	1	1

136AA	97	116	123	336
136AV	20	0	2	22
136VV	0	0	0	0

141LL	94	110	112	316
141LF	23	5	13	41
141FF	0	1	0	1

143 HH	63	110	106	279
143 HR	46	6	19	71
143 RR	8	0	0	8

154RR	106	114	98	318
154RH	11	2	26	39
154HH	0	0	1	1

171 QQ	55	13	32	100
171 QR	56	51	72	179
171 RR	6	52	21	79

### Frequency of OPP provirus among *PRNP *genotype

**Figure 2:**
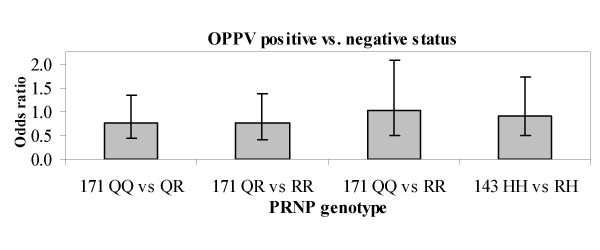
**Odds ratio and 95% confidence interval for effect of *PRNP *genotype upon frequency of OPPV positive animals**.

The presence or absence of OPPV provirus was compared among the *PRNP *171 and *PRNP *143 genotypes, using a statistical model accounting for age and breed, to determine if minor alleles within those genotypes affected the number of sheep that had detectable OPPV provirus. In the flock, 226 of 358 (63.1%) sheep had detectable OPPV provirus. Over half of the ewes were positive for OPPV provirus within each *PRNP *171 or 143 genotype (Table 2). The frequency of OPPV positive animals was not significantly different between the *171QQ*, *QR*, and *RR *genotypes as indicated by nominal and corrected p-values greater than 0.05 (Table 3) and equivalent odds ratios (Fig. 2). The 95% confidence intervals also indicate the range of potential effect sizes consistent with these data (Fig. 2). Also, the frequency of OPPV positive animals did not differ significantly between the *143HH *and *HR *genotypes.

**Table 2:** Number of ewes with (positive) or without (negative) detectable OPPV provirus among *PRNP *genotypes used for statistical comparison

	OPPV Provirus Status		% OPPV
*PRNP *genotype	negative	positive	positive
171 QQ	36	64	64.0
171 QR	61	118	65.9
171 RR	35	44	55.7
143 HH	103	176	63.1
143 HR	26	45	63.4

**Table 3:** Significance level for effect of *PRNP *genotype upon frequency of animals with detectable OPPV provirus

	OPPV positive vs negative p-value	
Genotype comparison	nominal	corrected
171 QQ vs. QR	0.23	0.90
171 QR vs. RR	0.23	0.90
171 QQ vs. RR	0.60	1.00
143 HH vs. RH	0.78	1.00

P-values are before (nominal, left) and after (corrected, right) step-down Bonferroni multiple test correction

### OPPV provirus levels among *PRNP *genotypes

**Figure 3:**
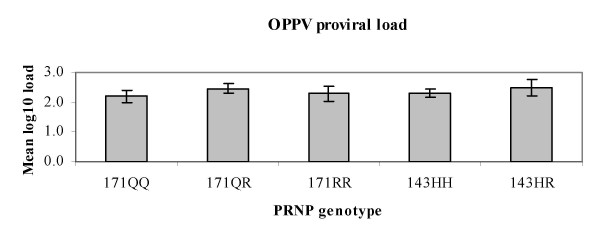
**Adjusted mean log10 provirus levels and 95% confidence interval among *PRNP *genotypes used for statistical comparison**.

The levels of OPPV provirus were compared among the *PRNP *171 and *PRNP *143 genotypes to determine whether particular genotypes were associated with higher or lower provirus levels once a ewe became infected. Adjusted mean log-transformed provirus levels with 95% confidence interval were equivalent among codon 171 and among codon 143 genotypes (Fig. 3). Adjusted mean log-transformed provirus levels were not significantly different among the *171QQ*, *QR*, and *RR *genotypes or among the *143HH *and *HR *genotypes in which nominal and corrected p-values were greater than 0.05 (Table 4).

**Table 4:** Significance level of OPPV proviral load levels between *PRNP *genotypes

	OPPV load p-value	
Genotype comparison	nominal	Corrected
171 QQ vs. QR	0.07	0.27
171 QR vs. RR	0.34	1.00
171 QQ vs. RR	0.60	1.00
143 HH vs. RH	0.27	1.00

p-values are before (nominal, left) and after (corrected, right) step-down Bonferroni multiple test correction

## Discussion

The present study was performed to determine if a *PRNP 171R *selection program impacts the presence or magnitude of OPPV infection. Allelic variation in *PRNP *could affect OPPV status if *PRNP *variants produce changes in PrP^c ^function or expression level relevant to OPPV, if *PRNP *is a pleiotropic gene, or if there are other molecules involved in prion pathogenesis that also affect OPPV pathogenesis. Alternatively, there may be nearby chromosomal regions affecting OPPV pathogenesis that are in linkage disequilibrium with certain *PRNP *alleles including, but not limited to, variants of *PRNP *promoter regions or *PRNP *homologues. However, the lack of association between *PRNP *genotype and OPPV status in this study indicates that the presence of a specific *PRNP *genotype does not influence the presence or magnitude of OPPV infection in this flock.

The study demonstrated that the frequency of sheep with detectable OPPV provirus was not related to the *PRNP 171R *(or *143R*) allele in an Idaho ewe flock. This suggests that it is no more likely that a *171RR *or *171QR *sheep within a flock would become infected when compared to a *171QQ *sheep. Likewise, the data suggest there is no difference in frequency of infection between the *143HH *and *143HR *sheep. Only ewes were sampled in this study so it is possible that introduction of rams could have a different affect, however it is unlikely considering that the frequency of OPPV in rams is equivalent, or perhaps lower than OPPV frequency in ewes [36,22].

Also, provirus levels in OPPV positive animals were not related to the *PRNP 171R *and *143R *alleles. Thus, *PRNP *selection should not affect progression of disease once animals become infected with OPPV. A shift of flock genetics to a greater frequency of *171QR *or *171RR *sheep is unlikely to accelerate shedding or transmission of OPPV. In these sheep there also was no difference in provirus levels between animals of the *143 HH *and *143HR *genotypes, thus there are no documented cases where PRNP genotypes impact OPPV infection.

Recent studies have shown that factors such as breed and age are important for OPPV, therefore all analyses in this study accounted for breed, age and differences in how each breed handled OPPV with age. For example, Rambouillet ewes are less likely to be positive for OPPV provirus than Columbia ewes and Rambouillet ewes can also better control OPPV provirus levels than either Columbia or Polypay ewes [23,37]. Further, these breed differences can change over time as some breeds show increasing provirus levels with age while others do not [37]. However, all the analyses in this study accounted for age and breed in the association models so that these factors would not influence tests for association with PRNP genotype.

Interactions between retrovirus' and normal or abnormal prion protein have been previously observed. The current findings do not exclude the possibility that increases in ovine PrP^c ^or CD230 expression could alter OPPV replication as observed in a human cell line where over-expression of human PrP^c ^thwarted HIV-1 replication [32]. OPPV replicates in mammary macrophages and microglia and transmits via ewe milk [38-40] and PrP^Sc ^is found in macrophages of lymphoid follicles and microglia and transmits via ewe milk [41-44,31] thereby suggesting functional overlap between host proteins involved in both prion and lentivirus pathogenesis. Additional links between prion and retrovirus' are indicated by data showing that caprine arthritis-encephalitis virus (CAEV) aids PrP^d ^accumulation in immortalized microglia *in vitro *[J Stanton, personal communication] and that scrapie infection increases MuLV expression and reciprocally MuLV accelerates scrapie pathogenesis [30].

This study is one of many examining *PRNP *selection effects. The *PRNP 171RR *genotype has no apparent effect on reproductive performance [45,46], ovulation rates and litter sizes [47], and only the Suffolk breed has lower lamb weaning weights [48]. Milk production and quality is not effected in Churra [49], East Friesian milk sheep [46] or Sardinian sheep and there are no significant changes in udder morphology [50]. Carcass and wool quality are not impaired [46,21] and *171R *may positively affect average daily gain [51]. *171R *has no effect on *Salmonella *resistance [21]. Finally, pedigree examination in Laxta Black Faced-type Navarra sheep showed no overall negative effect [52].

The present study taken together with previous investigations indicate that the correlated responses to *PRNP 171R *selection should be minimal. In total, ten different studies examining reproduction, meat, milk, fiber and infectious disease traits in a dozen different breeds found no overt negative effect from the *PRNP 171R *allele or *171RR *genotype. Additional studies may supplement present and previous results by examining other breeds, genotypes, retrovirus strains, diseases, environmental or management conditions, or production traits. This investigation of a flock with endemic OPPV shows that the frequency of OPPV infection and level of OPPV provirus loads are not affected by the *PRNP 171R *allele (occurring either in the 171QR heterozygous or 171RR homozygous genotypes) and supports *PRNP 171R *selection as a component of Scrapie control programs.

## Competing interests

The authors declare that they have no competing interests.

## Authors' contributions

RDH designed the study, performed sequence analysis, determined genotype distribution and frequencies, participated in statistical analysis, and drafted the manuscript. LHH participated in experimental design, developed and performed the RT-PCR assay, performed sequence analysis, and assisted in drafting the manuscript. SNW participated in experimental design, performed statistical analysis, and assisted in drafting the manuscript. KIOR participated in experimental design, performed sequence analysis, and provided editorial revisions to intellectual content. DPK participated in experimental design and provided editorial revisions to intellectual content. All authors read and approved the final manuscript.

## References

[B1] Bueler H Aguzzi A Sailer A Greiner RA Autenried P Aguet M Weissmann C Mice devoid of PrP are resistant to scrapie Cell 1993 73 1339 1347 10.1016/0092-8674(93)90360-3 8100741

[B2] Dickinson AG Meikle VM Fraser H Identification of a gene which controls the incubation period of some strains of scrapie agent in mice J Comp Pathol 1968 78 293 299 10.1016/0021-9975(68)90005-4 4970191

[B3] Bossers A de Vries R Smits MA Susceptibility of sheep for scrapie as assessed by in vitro conversion of nine naturally occurring variants of PrP J Virol 2000 74 1407 1414 10627551 10.1128/JVI.74.3.1407-1414.2000 PMC111475

[B4] Bossers A Schreuder BE Muileman IH Belt PB Smits MA PrP genotype contributes to determining survival times of sheep with natural scrapie J Gen Virol 1996 77 Pt 10 2669 2673 10.1099/0022-1317-77-10-2669 8887505

[B5] Hunter N Foster JD Goldmann W Stear MJ Hope J Bostock C Natural scrapie in a closed flock of Cheviot sheep occurs only in specific PrP genotypes Arch Virol 1996 141 809 824 10.1007/BF01718157 8678828

[B6] O'Rourke KI Holyoak GR Clark WW Mickelson JR Wang S Melco RP Besser TE Foote WC PrP genotypes and experimental scrapie in orally inoculated Suffolk sheep in the United States J Gen Virol 1997 78 Pt 4 975 978 9129673 10.1099/0022-1317-78-4-975

[B7] Westaway D Zuliani V Cooper CM Da Costa M Neuman S Jenny AL Detwiler L Prusiner SB Homozygosity for prion protein alleles encoding glutamine-171 renders sheep susceptible to natural scrapie Genes Dev 1994 8 959 969 10.1101/gad.8.8.959 7926780

[B8] Acutis PL Bossers A Priem J Riina MV Peletto S Mazza M Casalone C Forloni G Ru G Caramelli M Identification of prion protein gene polymorphisms in goats from Italian scrapie outbreaks J Gen Virol 2006 87 1029 1033 10.1099/vir.0.81440-0 16528054

[B9] Papasavva-Stylianou P Kleanthous M Toumazos P Mavrikiou P Loucaides P Novel polymorphisms at codons 146 and 151 in the prion protein gene of Cyprus goats, and their association with natural scrapie Vet J 2007 173 459 462 10.1016/j.tvjl.2005.09.013 16314132

[B10] Vaccari G Di Bari MA Morelli L Nonno R Chiappini B Antonucci G Marcon S Esposito E Fazzi P Palazzini N Identification of an allelic variant of the goat PrP gene associated with resistance to scrapie J Gen Virol 2006 87 1395 1402 10.1099/vir.0.81485-0 16603543

[B11] Hamir AN Gidlewski T Spraker TR Miller JM Creekmore L Crocheck M Cline T O'Rourke KI Preliminary observations of genetic susceptibility of elk (*Cervus elaphus nelsoni*) to chronic wasting disease by experimental oral inoculation J Vet Diagn Invest 2006 18 110 114 16566268 10.1177/104063870601800118

[B12] Johnson C Johnson J Clayton M McKenzie D Aiken J Prion protein gene heterogeneity in free-ranging white-tailed deer within the chronic wasting disease affected region of Wisconsin J Wildl Dis 2003 39 576 581 14567218 10.7589/0090-3558-39.3.576

[B13] O'Rourke KI Besser TE Miller MW Cline TF Spraker TR Jenny AL Wild MA Zebarth GL Williams ES PrP genotypes of captive and free-ranging Rocky Mountain elk (*Cervus elaphus nelsoni*) with chronic wasting disease J Gen Virol 1999 80 2765 2769 10573173 10.1099/0022-1317-80-10-2765

[B14] O'Rourke KI Spraker TR Hamburg LK Besser TE Brayton KA Knowles DP Polymorphisms in the prion precursor functional gene but not the pseudogene are associated with susceptibility to chronic wasting disease in white-tailed deer J Gen Virol 2004 85 1339 1346 10.1099/vir.0.79785-0 15105552

[B15] Bishop MT Hart P Aitchison L Baybutt HN Plinston C Thomson V Tuzi NL Head MW Ironside JW Will RG Predicting susceptibility and incubation time of human-to-human transmission of vCJD Lancet Neurol 2006 5 393 398 10.1016/S1474-4422(06)70413-6 16632309

[B16] Cervenakova L Goldfarb LG Garruto R Lee HS Gajdusek DC Brown P Phenotype-genotype studies in kuru: implications for new variant Creutzfeldt-Jakob disease Proc Natl Acad Sci USA 1998 95 13239 13241 9789072 10.1073/pnas.95.22.13239 PMC23768

[B17] McCormack JE Baybutt HN Everington D Will RG Ironside JW Manson JC PRNP contains both intronic and upstream regulatory regions that may influence susceptibility to Creutzfeldt-Jakob disease Gene 2002 288 139 146 10.1016/S0378-1119(02)00466-3 12034503

[B18] Zeidler M Stewart G Cousens SN Estibeiro K Will RG Codon 129 genotype and new variant CJD Lancet 1997 350 668 10.1016/S0140-6736(05)63366-1 9288076

[B19] O'Rourke KI Ovine scrapie. New tools for control of an old disease Vet Clin North Am Food Anim Pract 2001 17 283 300 vi 11515402

[B20] USDA Phase II Scrapie: Ovine Slaughter Surveillance Study 2002–2003 2003 USDA: APHIS: VS, CEAH, National Animal Health Monitoring System. Fort Collins, CO

[B21] Vitezica ZG Moreno CR Lantier F Lantier I Schibler L Roig A Francois D Bouix J Allain D Brunel JC Quantitative trait loci linked to PRNP gene controlling health and production traits in INRA 401 sheep Genet Sel Evol 2007 39 421 430 10.1051/gse:2007012 17612481 PMC2682820

[B22] Cutlip RC Lehmkuhl HD Sacks JM Weaver AL Seroprevalence of ovine progressive pneumonia virus in sheep in the United States as assessed by analyses of voluntarily submitted samples Am J Vet Res 1992 53 976 979 1320816

[B23] Herrmann-Hoesing LM White SN Lewis GS Mousel MR Knowles DP Development and validation of an ovine progressive pneumonia virus quantitative PCR Clin Vaccine Immunol 2007 14 1274 1278 17699832 10.1128/CVI.00095-07 PMC2168119

[B24] Brodie SJ Pearson LD Zink MC Bickle HM Anderson BC Marcom KA DeMartini JC Ovine lentivirus expression and disease. Virus replication, but not entry, is restricted to macrophages of specific tissues Am J Pathol 1995 146 250 263 7531949 PMC1870777

[B25] Arens M Use of probes and amplification techniques for the diagnosis and prognosis of human immunodeficiency virus (HIV-1) infections Diagn Microbiol Infect Dis 1993 16 165 172 10.1016/0732-8893(93)90016-Z 8467630

[B26] Verhofstede C Reniers S Van Wanzeele F Plum J Evaluation of proviral copy number and plasma RNA level as early indicators of progression in HIV-1 infection: correlation with virological and immunological markers of disease AIDS 1994 8 421 1427 10.1097/00002030-199410000-00008 7529507

[B27] Vitone F Gibellini D Schiavone P Re MC Quantitative DNA proviral detection in HIV-1 patients treated with antiretroviral therapy J Clin Virol 2005 33 194 200 15911440 10.1016/j.jcv.2004.11.003

[B28] Brodie SJ Marcom KA Pearson LD Anderson BC de la Concha-Bermejillo A Ellis JA DeMartini JC Effects of virus load in the pathogenesis of lentivirus-induced lymphoid interstitial pneumonia J Infect Dis 1992 166 531 541 1323619 10.1093/infdis/166.3.531

[B29] Zhang Z Watt NJ Hopkins J Harkiss G Woodall CJ Quantitative analysis of maedi-visna virus DNA load in peripheral blood monocytes and alveolar macrophages J Virol Methods 2000 86 13 20 10.1016/S0166-0934(99)00169-X 10713371

[B30] Lee KH Jeong BH Jin JK Meeker HC Kim JI Carp RI Kim YS Scrapie infection activates the replication of ecotropic, xenotropic, and polytropic murine leukemia virus (MuLV) in brains and spinal cords of senescence-accelerated mice: implication of MuLV in progression of scrapie pathogenesis Biochem Biophys Res Commun 2006 349 122 130 10.1016/j.bbrc.2006.08.016 16930537

[B31] Ligios C Sigurdson CJ Santucciu C Carcassola G Manco G Basagni M Maestrale C Cancedda MG Madau L Aguzzi A PrPSc in mammary glands of sheep affected by scrapie and mastitis Nat Med 2005 11 1137 1138 10.1038/nm1105-1137 16270061

[B32] Leblanc P Baas D Darlix JL Analysis of the interactions between HIV-1 and the cellular prion protein in a human cell line J Mol Biol 2004 337 1035 1051 10.1016/j.jmb.2004.02.007 15033368

[B33] Schneider DA Yan H Fry LM Alverson J White SN O'Rourke IK Myenteric neurons of the ileum that express somatostatin are a target of prion neuroinvasion in an alimentary model of sheep scrapie Acta Neuropathol 2008 115 651 661 10.1007/s00401-008-0374-2 18427817

[B34] Herrmann LM Hotzel I Cheevers WP On Top KP Lewis GS Knowles D Seven new ovine progressive pneumonia virus (OPPV) field isolates from Dubois Idaho sheep comprise part of OPPV clade II based on surface envelope glycoprotein (SU) sequences Virus Res 2004 102 215 220 10.1016/j.virusres.2004.02.001 15084404

[B35] Holm S A simple sequentially rejective bonferroni test procedure Scand J Stat 1979 6 65 70

[B36] Arsenault J Dubreuil P Girard C Simard C Belanger D Maedi-visna impact on productivity in Quebec sheep flocks (Canada) Prev Vet Med 2003 59 125 137 10.1016/S0167-5877(03)00086-2 12809758

[B37] Herrmann-Hoesing LM White SN Mousel MR Lewis GS Knowles DP Ovine progressive pneumonia provirus levels associate with breed and Ovar-DRB1 Immunogenetics 2008 60 12 749 758 18797863 10.1007/s00251-008-0328-9

[B38] Carrozza ML Mazzei M Bandecchi P Arispici M Tolari F In situ PCR-associated immunohistochemistry identifies cell types harbouring the Maedi-Visna virus genome in tissue sections of sheep infected naturally J Virol Methods 2003 107 121 127 10.1016/S0166-0934(02)00208-2 12505625

[B39] Ebrahimi B Allsopp TE Fazakerley JK Harkiss GD Phenotypic characterisation and infection of ovine microglial cells with Maedi-Visna virus J Neurovirol 2000 6 320 328 10.3109/13550280009030758 10951556

[B40] Herrmann-Hoesing LM Palmer GH Knowles DP Evidence of proviral clearance following postpartum transmission of an ovine lentivirus Virology 2007 362 226 234 10.1016/j.virol.2006.12.021 17267002

[B41] Andreoletti O Levavasseur E Uro-Coste E Tabouret G Sarradin P Delisle MB Berthon P Salvayre R Schelcher F Negre-Salvayre A Astrocytes accumulate 4-hydroxynonenal adducts in murine scrapie and human Creutzfeldt-Jakob disease Neurobiol Dis 2002 11 386 393 10.1006/nbdi.2002.0558 12586548

[B42] Caplazi P O'Rourke K Wolf C Shaw D Baszler TV Biology of PrPsc accumulation in two natural scrapie-infected sheep flocks J Vet Diagn Invest 2004 16 489 496 15586562 10.1177/104063870401600601

[B43] Herrmann LM Cheevers WP Davis WC Knowles DP O'Rourke KI CD21-positive follicular dendritic cells: A possible source of PrP(Sc) in lymph node macrophages of scrapie-infected sheep Am J Pathol 2003 162 1075 1081 12651600 10.1016/S0002-9440(10)63904-1 PMC1851249

[B44] Konold T Moore SJ Bellworthy SJ Simmons HA Evidence of scrapie transmission via milk BMC Vet Res 2008 4 14 18397513 10.1186/1746-6148-4-14 PMC2374774

[B45] Alexander BM Stobart RH Moss GE Scrapie resistance and production traits in Rambouillet rams: Ram performance test 2002–2006 Res Vet Sci 2008 85 2 345 348 10.1016/j.rvsc.2007.11.004 18093625

[B46] De Vries F Hamann H Drogemuller C Ganter M Distl O Analysis of associations between the prion protein genotypes and production traits in East Friesian milk sheep J Dairy Sci 2005 88 392 398 15591404 10.3168/jds.S0022-0302(05)72699-0

[B47] Sweeney T Hanrahan JP O'Doherty E Is there a relationship between prion protein genotype and ovulation rate and litter size in sheep? Anim Reprod Sci 2007 101 153 157 10.1016/j.anireprosci.2006.12.004 17204381

[B48] Alexander BM Stobart RH Russell WC O'Rourke KI Lewis GS Logan JR Duncan JV Moss GE The incidence of genotypes at codon 171 of the prion protein gene (PRNP) in five breeds of sheep and production traits of ewes associated with those genotypes J Anim Sci 2005 83 455 459 15644519 10.2527/2005.832455x

[B49] Alvarez L Gutierrez-Gil B San Primitivo F de la Fuente LF Arranz JJ Influence of prion protein genotypes on milk production traits in Spanish Churra sheep J Dairy Sci 2006 89 1784 1791 16606750 10.3168/jds.S0022-0302(06)72247-0

[B50] Salaris S Casu S Carta A Investigating the relationship between the prion protein locus and udder morphology traits and milk yield in Sardinian sheep J Anim Sci 2007 85 2840 2845 10.2527/jas.2006-610 17526657

[B51] Evoniuk JM Berg PT Johnson ML Larson DM Maddock TD Stoltenow CL Schauer CS O'Rourke KI Redmer DA Associations between genotypes at codon 171 and 136 of the prion protein gene and production traits in market lambs Am J Vet Res 2007 68 1073 1078 10.2460/ajvr.68.10.1073 17916013

[B52] Alfonso L Parada A Legarra A Ugarte E Arana A The effects of selective breeding against scrapie susceptibility on the genetic variability of the Latxa Black-Faced sheep breed Genet Sel Evol 2006 38 495 511 10.1051/gse:2006017 16954042 PMC2689259

